# Supplementary material for: A dual role of Cohesin in DNA DSB repair
Source: Nat Commun. 2025 Jan 20;16:843. doi: 10.1038/s41467-025-56086-4 (PMC11747280; doi:10.1038/s41467-025-56086-4)
Supplement: Supplementary file 1 — Supplementary Information [file 41467_2025_56086_MOESM1_ESM.pdf]

Supplementary Figures

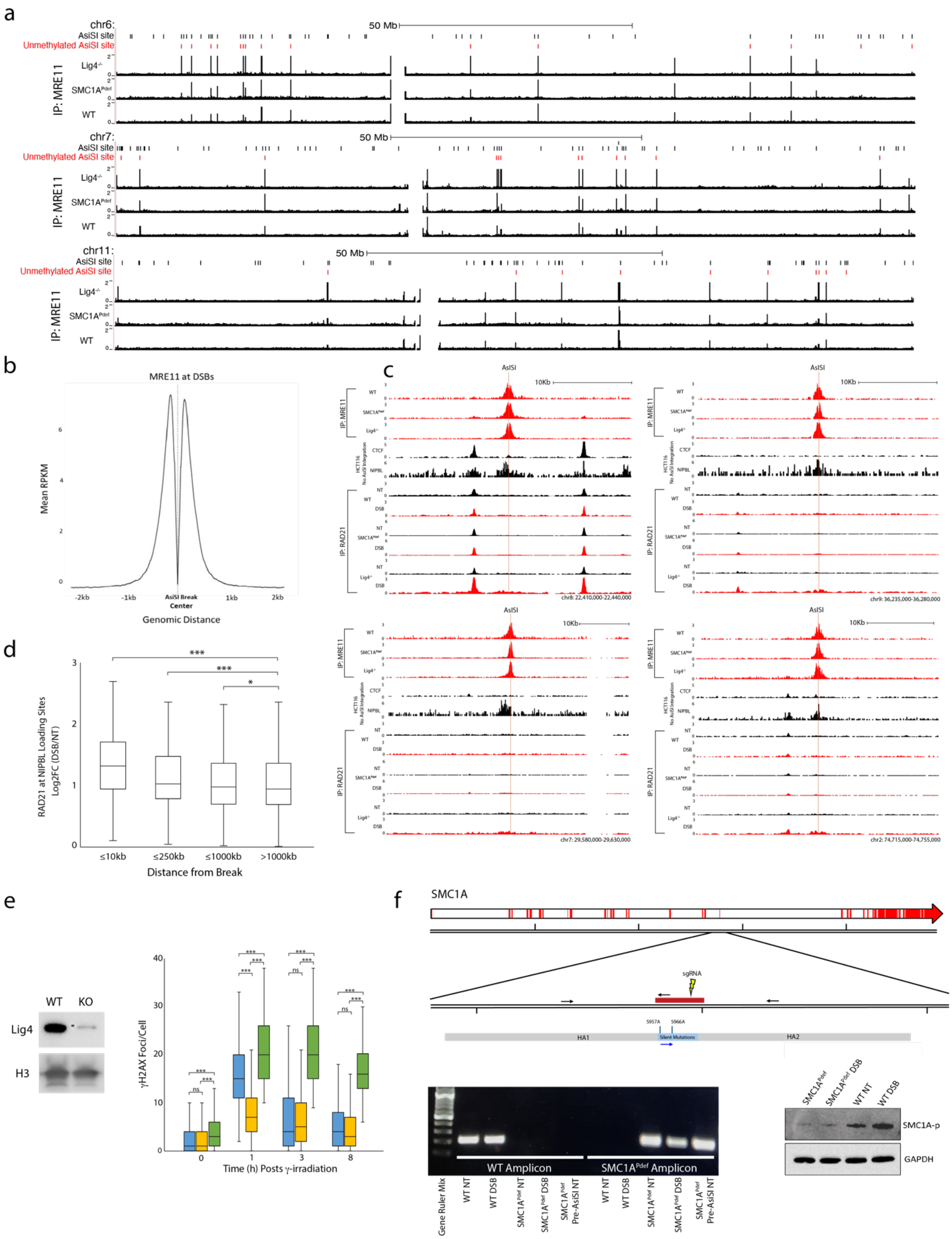

Supplementary Figure 1: ChIPseq and HiC were used to study changes in genome architecture after AsiSI DSB induction. **a)** MRE11 breaks were consistent among genetic backgrounds as shown in three full chromosome examples. MRE11 signal localized primarily to unmethylated AsiSI sites (red). **b)** Composite view of MRE11 signal at AsiSI breaks. **c)** Four strong DSB sites are shown at 30-50kb genomic resolution. Conditions in which damage is induced are shown in red. We did not observe significant RAD21 peaks at double strand ends. **d)** Enrichment of cohesin measured at NIPBL loading sites within distance from break categories ≤10, ≤100, ≤1Mb (n =93, 510, 1128) versus >1Mb (n = 17207; P = 2E-8, 9E-8, 0.02). The number of asterisks assigned to a given item indicate the level of significance *P<0.05, *P<0.01 ***P<0.001 (two sample, paired; T-test). Box plot parameters: The lower and upper limits of the boxes in the plot represent the 25th and the 75th quartiles respectively. The central line represents the median, while each whiskers represent one IQR. **e)** Confirmation of Cas9-mediated deletion of Ligase 4 by western blot (left). The diminished repair phenotype expected in G1 cells was observed by measuring γH2AX foci over a time course: 0, 1, 3, and 8h post treatment with 2.5Gy γ-irradiation in WT (n = 345, 457, 427, 377), ATMi (n = 91, 482, 410, 491), and LIG4$^{/-}$ (n = 197, 215, 186, 138) cells. At the 8h time point, the lack of repair is clear in LIG4$^{-/-}$cells compared to WT cells (P >1E-16). Box plot and statistical parameters are the same as panel d. **f)** Schematic for design of SMC1A^{Pdef} mutant (upper panel) with PCR genotyping validation of homozygous SMC1A^{Pdef} versus WT (lower let) and western blot confirming CRISPR mediated gene editing (lower right). Source data for all panels are provided as a Source Data file.

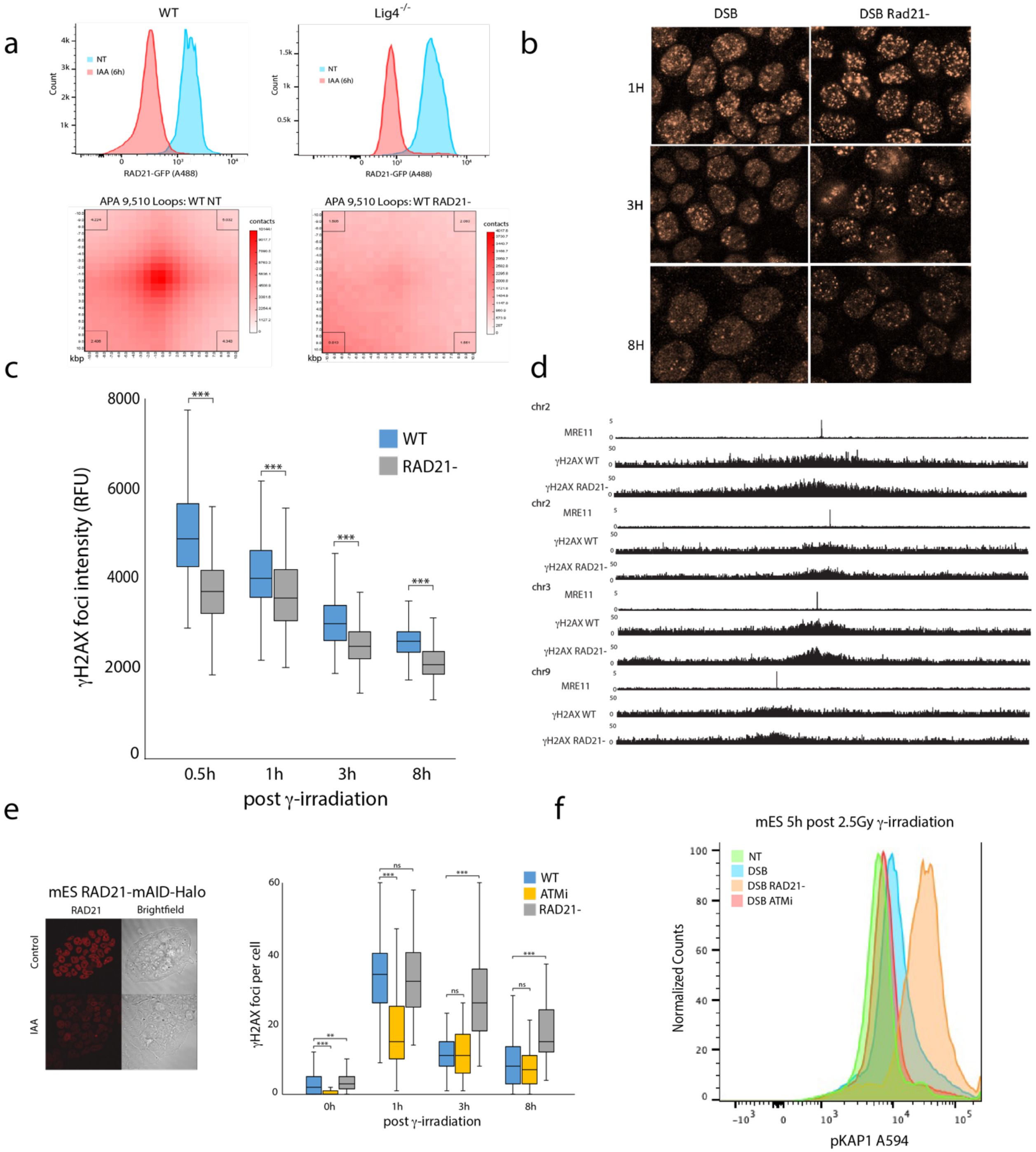

Supplementary Figure 2: DNA repair assays were replicated in complementary systems. a) RAD21 was efficiently degraded by the addition of indole 3 acetic acid (IAA) for 6h (top panels) measured via loss of RAD21-GFP signal. Inactivation of the cohesin complex was confirmed by loss of genomic loops visualized by APA analyses (bottom panels). **b)** Representative images from γH2AX repair assays in HCT116 cells. **c)** Foci intensities were measured following treatment

with 2.5Gy of γ-irradiation in the presence or absence of cohesin at 0.5h (n = 332, 413), 1h (n = 467, 433), 3h (n = 427, 303), and 8h (n = 377, 342) timepoints. Agilent BioTek software was used to process images and count foci and measure intensity. The number of asterisks assigned to a given item indicate the level of significance *P<0.05, *P<0.01 ***P<0.001 (two sample, paired; T-test). Box plot parameters: The lower and upper limits of the boxes in the plot represent the 25th and the 75th quartiles respectively. The central line represents the median, while each whiskers represent one IQR. **d)** Genome browser view of γH2AX domains in the presence or absence of cohesin. MRE11 tracks identify exact break position **e)** The left panel shows an example of RAD21 depletion in mES cells treated with 750μM IAA for 6h. Since RAD21 is fused to a Halo tag, these cells were imaged with the Janelia Fluro 549 ligand. The right panel shows γH2AX repair assays performed in mouse ES cells following treatment with 2.5Gy γ-irradiation at 0h, 1h, 3h, and 5h in WT (n = 141, 166, 164, 121), RAD21- (n = 50, 110, 126, 81)and ATMi (n = 56, 173, 122, 149) conditions. Box plot and statistical parameters are the same as panel c. **f)** DNA repair was quantified in mES cells by measuring the phosphorylation of ATM substrate, Kap1, 5h post γ-irradiation using flow cytometry. Source data for all panels are provided as a Source Data file.

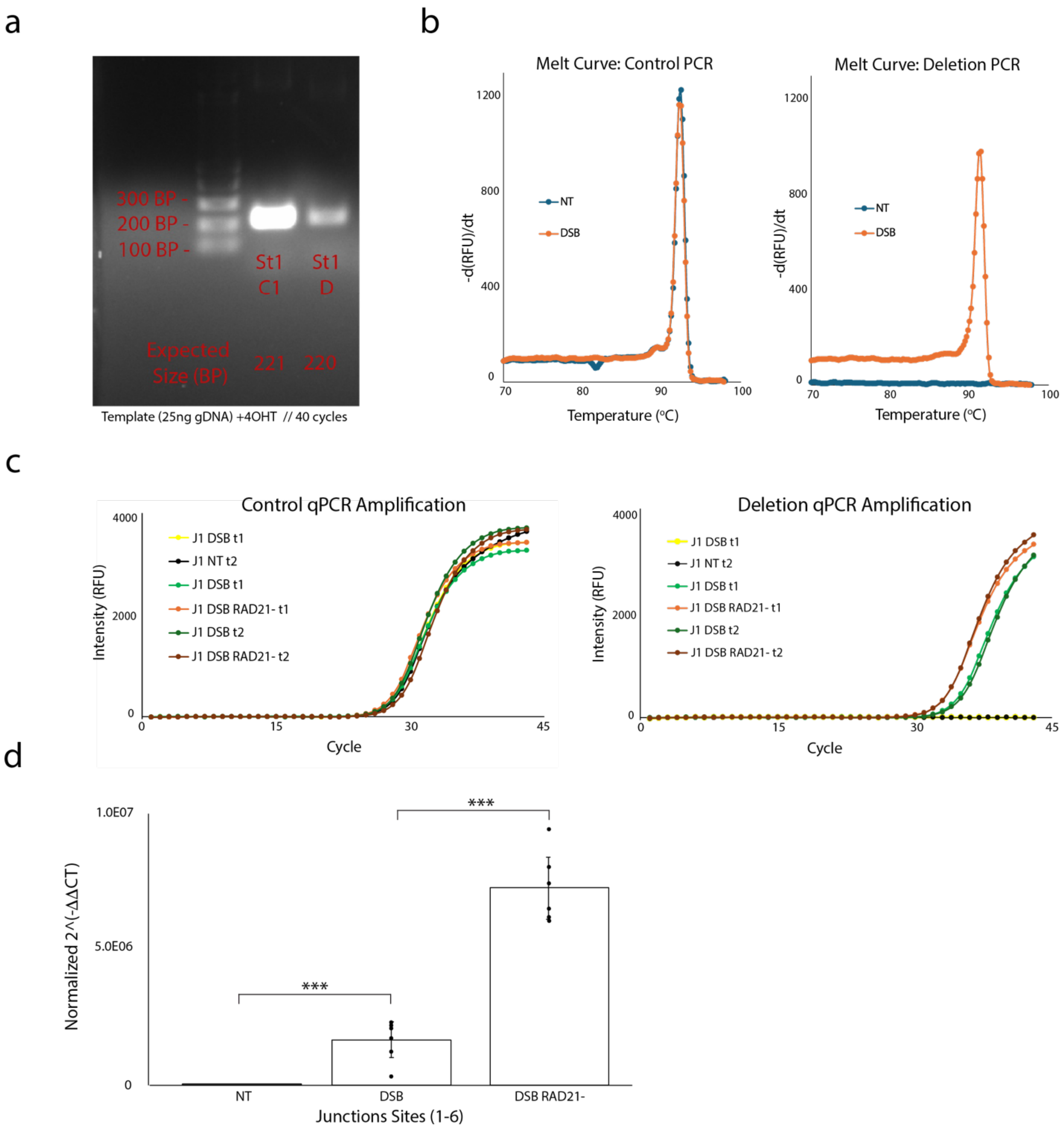

Supplementary Figure 3: In depth analysis of qPCR cis deletion detection and cell cycle controls. a) An example PCR validation of deletion junction 1 (J1) using agarose electrophoresis 1 **b)** An example melting curves for J1 in the presence and absence of DNA damage show specific single product amplification for cis deletions. **c)** Representative amplification curves for two technical replicates using J1 control and deletion primers. **d)** Deletion junctions were measured for by qPCR after pre-treatment with G1 inhibitor Palbociclib (Palb) for 6h prior to 72h AsiSI induction to ensure complete cell cycle arrest. Each dot represents a junction sites (n =

6), while error bars represent SD between junction sites for a given condition. The number of asterisks assigned to a given item indicate the level of significance *P<0.05, *P<0.01 ***P<0.001 (one tailed, paired T-test). Source data for all panels are provided as a Source Data file.

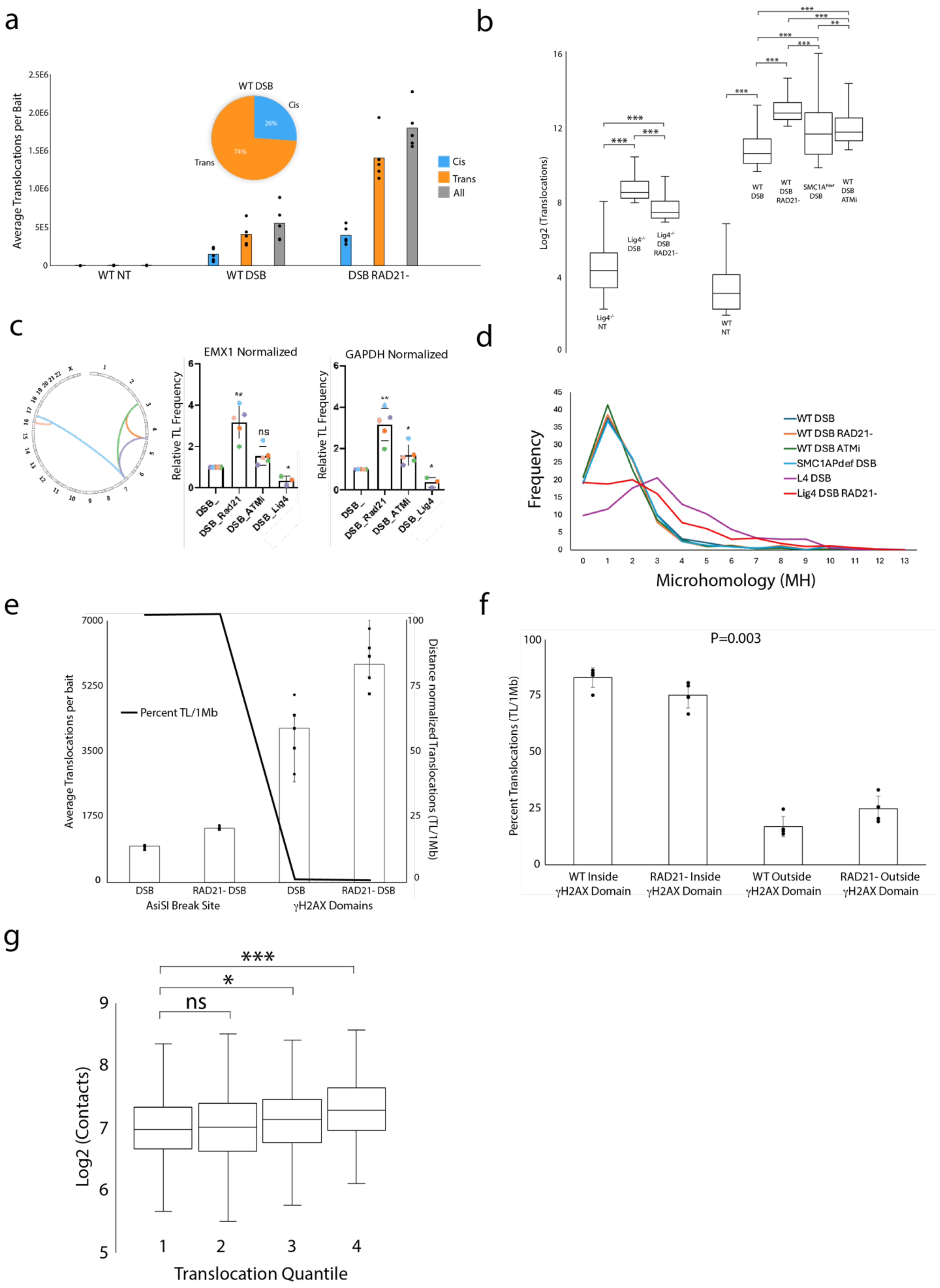

a
WT DSB
Cis
Trans
26%
74%
Average Translocations per Bait
2.5E6
2.0E6
1.5E6
1.0E6
5E5
0
Cis
Trans
All
WT NT
WT DSB
DSB RAD21-
b
Log2 (Translocations)
16
12
8
4
0
***
**
Lig4-/- NT
Lig4-/- DSB
Lig4-/- DSB RAD21-
WT NT
WT DSB
WT DSB RAD21-
SMC1APdef DSB
WT DSB ATMi
c
EMX1 Normalized
GAPDH Normalized
Relative TL Frequency
ns
DSB_
DSB_Rad21
DSB_ATMi
DSB_Lig4
d
Frequency
Microhomology (MH)
WT DSB
WT DSB RAD21-
WT DSB ATMi
SMC1APdef DSB
L4 DSB
Lig4 DSB RAD21-
e
Average Translocations per bait
Distance normalized Translocations (TL/1Mb)
Percent TL/1Mb
7000
5250
3500
1750
0
100
75
50
25
DSB
RAD21- DSB
AsiSI Break Site
γH2AX Domains
f
P=0.003
Percent Translocations (TL/1Mb)
WT Inside γH2AX Domain
RAD21- Inside γH2AX Domain
WT Outside γH2AX Domain
RAD21- Outside γH2AX Domain
g
***
*
ns
Log2 (Contacts)
9
8
7
6
5
1
2
3
4
Translocation Quantile

Supplementary Figure 4: Global analysis of HTGTS data a) Equivalence of cis and trans end joining events in the presence (DSB) and absence of RAD21 (DSB RAD21-) compared measured by total (bar graphs) and percentages for DSB (pie charts). Each dot represents a bait site (n = 5), while error bars represent SD between junction sites for a given condition. **b)** Box plots show log2 of translocations for the top 500 unique translocations in each condition. The number of asterisks assigned to a given item indicate the level of significance *P<0.05, *P<0.01 ***P<0.001 (one tailed, paired; T-test). Box plot parameters: The lower and upper limits of the boxes in the plot represent the 25th and the 75th quartiles respectively. The central line represents the median, while each whiskers represent one IQR. **c)** To verify the HTGTS results, 5 translocations were selected and screened using qPCR to verify the impact of cohesin depletion on increased translocation frequency (left panel). WT cells showed an Increase in translocation frequency in the absence of RAD21 evidenced by normalization using *EMX1* (middle) and GAPDH (right) compared to RAD21 replete cells, consistent with HTGTS. Each dot represents a tested translocation site (n = 5), while error bars represent SD between junction sites for a given condition. **d)** Microhomology usage was calculated for all backgrounds for non-AsiSI junctions in the presence or absence of cohesin. **e)** Average translocations per bait and percent translocations per bait are displayed on the left and right axes respectively. Data represented as bars correspond to the average translocation axis (left axis). Each dot represents a bait site (n = 5), while error bars represent SD between junction sites for a given condition. Data represented as a line correspond to percent translocation per 1Mb axis (right axis). **f)** The distribution of translocations occurring inside and outside of γH2AX domains was measured in the presence and absence of cohesin. Statistical analysis via T-test yields a single P value both comparisons between WT and RAD21- (P = 0.003). Each dot represents a tested translocation site (n = 5), while error bars represent SD between junction sites for a given condition. **g)** Translocations were broken into four quantiles based on intensity and contact frequencies for these quantiles were compared (n = 257 per quantile). Translocation quantiles 3 and 4 (P = 0.03, 4E-8) were significantly enriched for interactions compared with quantile 1. Box plot and statistical parameters are the same as panel b. Source data for all panels are provided as a Source Data file.

Supplementary Table 1: A complete list of kits and chemicals used in this study. Accompanying commercial sources and product identifiers are also included.

**Material**	**Source**	**Identifier**
Indole-3-acetic acid (IAA)	Millipore Sigma	I5148
Cell Line Nucleofector™ Kit V	Lonza	VCA-1003
Lipofectamine™ LTX Reagent with PLUS™ Reagent	ThermoFisher Scientific	A12621
4-Hydroxytamoxifen (4OHT)	Millipore Sigma	SML1666
ATMi KU-55933	Fisher Scientific	35-441-0
Hoechst 33342	ThermoFisher Scientific	62249
m-slides (ibitreat)	Ibidi	80826
Paraformaldehyde (PFA)	Electron Microscopy Sciences	15710
BD Perm/Wash Buffer	BD Bioscience	554723
Comet Assay Kit	Abcam	ab238544
Dynabeads™ MyOne™ Streptavidin T1	ThermoFisher Scientific	65601
Q5® High-Fidelity DNA Polymerase	New England Biolabs	M0491S
PowerUp SYBR Green Master Mix	ThermoFisher Scientific	A25780
cOmplete™, Mini, EDTA-free Protease Inhibitor Cocktail	Millipore Sigma	11836170001
Dynabeads™ Protein A	ThermoFisher Scientific	10002D
Ovation® Ultralow System V2	Tecan	M01379

Supplementary Table 2: A complete list of oligos used in this study is provided. All Oligos were purchased through Sigma Aldrich.

DNA Oligo Table:

Lig4 sgRNA N’<br>aaacCTCGGGTGTCGCCGGCAAATc<br>caccgATTTGCCGGCGACACCCGAG
Lig4 sgRNA C’<br>aaacTGTCGATGGTTTTAGCTAAAc<br>caccgTTTAGCTAAAACCATCGACA
Smc1A sgRNA<br>caccGGCCTCTCGTGCATAGATAC<br>aaacGTATCTATGCACGAGAGGCC
Lig4 Screen F:<br>GGCTTCAGTTTGCTTCCC
Lig4 Screen R (WT):<br>GAGAGGGGCCCTAACGAG
Lig4 Screen R (Del):<br>GCTCCATGAAACCGAAGCTC
SMC1A WT Screen F:<br>GCACCTGGCCATAGAGCTC<br>SMC1A WT Screen R:<br>GAACCACTCACTGAGTCCTCC
SMC1A Pdef Screen F:<br>CTGCACAAGGTGAAGATTCGG<br>SMC1A Pdef Screen R:<br>GGAGATCCTGCCTCACAACT
qPCR S1 F<br>GACAGCCCAAGCCGTAGAAGC
qPCR S1 R (WT)<br>GCAGCTGCAACAACCAGTGC
qPCR S1 R (Del)<br>GGCGACTCTCGGCAAATTCG
qPCR S2 F<br>GCAGCAGCTTCCCGGGCTTTG
qPCR S2 R (WT)<br>CGGCCCTAGCGACCCGAGTC
qPCR S2 R (Del)<br>GGGGCACTCCTGATGCCTAC
qPCR S3 F<br>GGAGAATTCCTCACTGTAGGC
qPCR S3 R (WT)<br>CAGAGAGGAATCCGCACCCTC
qPCR S3 R (Del)<br>CCTGCGTCAGGTTGTAGGCT
qPCR S4 F

GTAATGTTGCTTGTCTGATTAG
qPCR S4 R (WT)<br>GCTGAGGCAGGGGAATCGC
qPCR S4 R (Del)<br>CACAGCTGTCTGTTTCACGTG
qPCR S5 F<br>CAGGTGGAGGAAGCAGTCGC
qPCR S5 R (WT)<br>CCGACGTCCTCGGCCTGC
qPCR S5 R (Del)<br>CAACCCAGGGACTACATCTC
qPCR S6 F<br>CCAGCGCAGACCTATGAATG
qPCR S6 R (WT)<br>GTGGTGCTTAGCCCAGGAC
qPCR S6 R (Del)<br>CCAGACTGGCGAGGAGGAG
HTGTS S1_bio_R<br>/5Bio/CACCTCTTTGCGCAGGAAGATC
HTGTS S1_red_R<br>CCTGATAGCCGTAGAAGTTGGG
HTGTS S2_bio_F<br>/5Bio/GATGGGGAAGAAACGGTTGG
HTGTS S2_red_F<br>GAGGCCAAGAAATGCACACG
HTGTS S3_bio_F<br>/5Bio/GCTTCGGTTGCATTCTTAAAG
HTGTS S3_red_F<br>CAGAGCAGAAACGGCACGAG
HTGTS S4_bio_F<br>/5Bio/GTGTTGCGGTTTCGGGGCGC
HTGTS S4_red_F<br>CCTCCCGTAGGCGATCAGAC
HTGTS S5_bio_F<br>/5Bio/GCGTCTACCCCGAGCGTCCG
HTGTS S5_red_F<br>GACTTCCCGCCCCTAGGCTG

Supplementary Table 3: A complete list of antibodies used in this study. Accompanying commercial sources and product identifiers are also included.

**Antibody**	**Source**	**Identifier**
Rad21 (ChIP)	Abcam	Ab992
MRE11 (ChIP)	Novus Biologicals	NB100-142
γH2AX (ChIP)	Abcam	Ab81299
γH2AX-A555 (IF)	Millipore Sigma	05-636-AF555
Rabbit anti-Phospho KAP-1 (S824)	Bethyl Laboratories	A300-767A
Recombinant Anti-SMC1A (pS957) (WB)	Abcam	ab137871
DNA Ligase IV (WB)	Cell Signaling Technology	14649S
