# Supplementary material for: A dual role of Cohesin in DNA DSB repair
Source: Nat Commun. 2025 Jan 20;16:843. doi: 10.1038/s41467-025-56086-4 (PMC11747280; doi:10.1038/s41467-025-56086-4)
Supplement: Supplementary file 2 — Description of Additional Supplementary Files [file 41467_2025_56086_MOESM2_ESM.pdf]

<u>Description of Additional Supplementary Files:</u>

Supplementary Data 1: This Microsoft Excel file contains a single sheet that lists the genomic positions of MRE11 peaks observed for all treatments and backgrounds in ChIPseq experiments. The following format is followed for denoting a peak- genomic position 1[left most boundary of peak]-2[right most boundary of peak].

Supplementary Data 2: This Microsoft Excel file contains a single sheet that lists the genomic positions of RAD21 peaks observed for all treatments and backgrounds in ChIPseq experiments. The following format is followed for denoting a peak- genomic position 1[left most boundary of peak]-2[right most boundary of peak].

Supplementary Data 3: This Microsoft Excel file contains a single sheet that lists the genomic positions of common, gained, and lost loops between NT and DSB. The following format is followed for denoting a loop- genomic position 1-2 [this defines the boundaries for the left loop anchor] // genomic positions 3-4 [this defines the boundaries for the right loop anchor]. Gained and lost loops within <1Mb of loop anchors are shown in adjacent columns (yes/no) and are highlighted in yellow.
