# Supplementary material for: A dual role of Cohesin in DNA DSB repair
Source: Nat Commun. 2025 Jan 20;16:843. doi: 10.1038/s41467-025-56086-4 (PMC11747280; doi:10.1038/s41467-025-56086-4)
Supplement: Supplementary file 6 — Reporting Summary [file 41467_2025_56086_MOESM6_ESM.pdf]

nature portfolio

Corresponding author(s): Michael Fedkenheuer

Last updated by author(s): Dec 17, 2024

Reporting Summary

Nature Portfolio wishes to improve the reproducibility of the work that we publish. This form provides structure for consistency and transparency in reporting. For further information on Nature Portfolio policies, see our Editorial Policies and the Editorial Policy Checklist.

Statistics

For all statistical analyses, confirm that the following items are present in the figure legend, table legend, main text, or Methods section.

n/a	Confirmed	
☐	☒	The exact sample size (*n*) for each experimental group/condition, given as a discrete number and unit of measurement
☐	☒	A statement on whether measurements were taken from distinct samples or whether the same sample was measured repeatedly
☐	☒	The statistical test(s) used AND whether they are one- or two-sided<br>*Only common tests should be described solely by name; describe more complex techniques in the Methods section.*
☐	☒	A description of all covariates tested
☐	☒	A description of any assumptions or corrections, such as tests of normality and adjustment for multiple comparisons
☐	☒	A full description of the statistical parameters including central tendency (e.g. means) or other basic estimates (e.g. regression coefficient) AND variation (e.g. standard deviation) or associated estimates of uncertainty (e.g. confidence intervals)
☐	☒	For null hypothesis testing, the test statistic (e.g. *F*, *t*, *r*) with confidence intervals, effect sizes, degrees of freedom and *P* value noted<br>*Give P values as exact values whenever suitable.*
☒	☐	For Bayesian analysis, information on the choice of priors and Markov chain Monte Carlo settings
☐	☒	For hierarchical and complex designs, identification of the appropriate level for tests and full reporting of outcomes
☒	☐	Estimates of effect sizes (e.g. Cohen's *d*, Pearson's *r*), indicating how they were calculated

Our web collection on statistics for biologists contains articles on many of the points above.

Software and code

Policy information about availability of computer code

Data collection	This has been clarified in the methods
Data analysis	This has been clarified in the methods

For manuscripts utilizing custom algorithms or software that are central to the research but not yet described in published literature, software must be made available to editors and reviewers. We strongly encourage code deposition in a community repository (e.g. GitHub). See the Nature Portfolio guidelines for submitting code & software for further information.

Data

Policy information about availability of data

All manuscripts must include a data availability statement. This statement should provide the following information, where applicable:

- Accession codes, unique identifiers, or web links for publicly available datasets
- A description of any restrictions on data availability
- For clinical datasets or third party data, please ensure that the statement adheres to our policy

ChIPseq and HiC data generated in this study have been deposited in the Geo database under accession code GSE250510 https://www.ncbi.nlm.nih.gov/geo/query/acc.cgi?acc=GSE250510. The processed ChIPseq and HiC data are provided in the Supplementary Information/Source Data file. The HTGTS data used in this study are available in the SRA database under accession code PRJNA1087267 https://www.ncbi.nlm.nih.gov/bioproject/PRJNA1087267. The processed HTGTS data are

provided in the Supplementary Information/Source Data file. Uncropped gel images and all data pertinent to the figures is provided in the Supplementary Information/Source Data file.

Research involving human participants, their data, or biological material

Policy information about studies with human participants or human data. See also policy information about sex, gender (identity/presentation), and sexual orientation and race, ethnicity and racism.

Reporting on sex and gender	N/A
Reporting on race, ethnicity, or other socially relevant groupings	N/A
Population characteristics	N/A
Recruitment	N/A
Ethics oversight	N/A

Note that full information on the approval of the study protocol must also be provided in the manuscript.

Field-specific reporting

Please select the one below that is the best fit for your research. If you are not sure, read the appropriate sections before making your selection.

☒ Life sciences ☐ Behavioural & social sciences ☐ Ecological, evolutionary & environmental sciences

For a reference copy of the document with all sections, see nature.com/documents/nr-reporting-summary-flat.pdf

Life sciences study design

All studies must disclose on these points even when the disclosure is negative.

Sample size	Sample sizes are now clearly defined in the figure legends.
Data exclusions	MRE11 peaks at non-AsiSI sites were excluded from analysis. This is specified in the methods and results.
Replication	Complete information about replication is now included in the figure legends.
Randomization	Randomization was not required.
Blinding	Experiments were not blinded. Cell line validation was blind between laboratories.

Reporting for specific materials, systems and methods

We require information from authors about some types of materials, experimental systems and methods used in many studies. Here, indicate whether each material, system or method listed is relevant to your study. If you are not sure if a list item applies to your research, read the appropriate section before selecting a response.

Materials & experimental systems

n/a	Involved in the study	
☐	☒	Antibodies
☐	☒	Eukaryotic cell lines
☒	☐	Palaeontology and archaeology
☒	☐	Animals and other organisms
☒	☐	Clinical data
☒	☐	Dual use research of concern
☒	☐	Plants

Methods

n/a	Involved in the study	
☐	☒	ChIP-seq
☐	☒	Flow cytometry
☒	☐	MRI-based neuroimaging

Antibodies

Antibodies used	All antibodies used are now described in the methods section with product identifiers. The antibodies used are now all listed in the supplementary resource table 3.

Validation	These are commercial antibodies. Product numbers and providers for each antibody are documented.

Eukaryotic cell lines

Policy information about cell lines and Sex and Gender in Research

Cell line source(s)	Sources for materials used are cited in the manuscript text
Authentication	All cell lines used were rigorously validated and continuously validated for each experiment. (ie loss of RAD21 was confirmed by FACs and DSB induction was confirmed by either gH2AX foci or MRE11 ChIPseq for all experiments and replicates)
Mycoplasma contamination	All cell lines tested negative for mycoplasm
Commonly misidentified lines (See ICLAC register)	No commonly misidentified cell lines were used in the study

Plants

Seed stocks	N/A
Novel plant genotypes	N/A
Authentication	N/A

ChIP-seq

Data deposition

☒ Confirm that both raw and final processed data have been deposited in a public database such as GEO.

☒ Confirm that you have deposited or provided access to graph files (e.g. BED files) for the called peaks.

Data access links *May remain private before publication.*	ChIPseq and Hi-C data has been deposited to Geo: GSE250510 Reviewer token is: azglqqgadlcvxqp<br>HTGTS data has been deposited to SRA: Accession to cite for these SRA data: PRJNA1087267 Temporary Submission ID: SUB14306537. These accessions are now public.
Files in database submission	DATA Provided In Geo
Genome browser session (e.g. UCSC)	DATA Provided In Geo

Methodology

Replicates	Details included in the methods section
Sequencing depth	Included in methods section
Antibodies	Included at the end of the resource table and methods section
Peak calling parameters	Included in the methods.
Data quality	With all experiments and data sets generated from those experiments, we begin by determining signal to noise ratio to determine our sensitivity and necessary sample sizes. This includes the basic positive and negative thresholding controls. All experiments are then checked for DNA damage and cohesin depletion for every biological replicate. We expand this further by performing and processing each biological replicate in 1 batch whenever possible. When this was not possible, samples were processed with a WT cell line in parallel for normalization. Number or biological replicates are specified in the text.
Software	Software utilized is described in the methods in more detail.

Flow Cytometry

Plots

Confirm that:

☒ The axis labels state the marker and fluorochrome used (e.g. CD4-FITC).

☒ The axis scales are clearly visible. Include numbers along axes only for bottom left plot of group (a 'group' is an analysis of identical markers).

☒ All plots are contour plots with outliers or pseudocolor plots.

☒ A numerical value for number of cells or percentage (with statistics) is provided.

Methodology

Sample preparation	Detailed in the Methods
Instrument	Detailed in the Methods
Software	Detailed in the Methods
Cell population abundance	Detailed in the Methods
Gating strategy	Basic single cell gating

☐ Tick this box to confirm that a figure exemplifying the gating strategy is provided in the Supplementary Information.
